# The Value of Multimodal Imaging in Early Phenotyping of Cardiomyopathies: A Family Case Report

**DOI:** 10.3390/jpm13050742

**Published:** 2023-04-27

**Authors:** Maria Livia Iovănescu, Diana Ruxandra Hădăreanu, Sebastian Militaru, Cristina Florescu, Constantin Militaru, Ionuț Donoiu

**Affiliations:** 1 Department of Cardiology, University of Medicine and Pharmacy of Craiova, 200349 Craiova, Romania; 2 Clinical Emergency County Hospital of Craiova, 200642 Craiova, Romania; 3 Filantropia Clinical Hospital, 200516 Craiova, Romania

**Keywords:** hypokinetic non-dilated cardiomyopathy, left ventricular non-compaction, titin mutation, imaging

## Abstract

Cardiomyopathies are structural and functional myocardial disorders that are not caused by other specific conditions such as coronary artery disease, arterial hypertension, valvular disease or congenital heart diseases. They are grouped into specific morphological and functional phenotypes, and sub-classified into familial and non-familial forms, with the dilated phenotype being the most frequent. However, there are many overlapping features between these phenotypes, complicating the diagnosis and management of patients. We report here the case of three related patients with different types of cardiomyopathies, emphasizing the importance of a multimodal approach to diagnosis.

## 1. Introduction

Dilated cardiomyopathy (DCM) is traditionally defined by left ventricular (LV) or biventricular dilation and systolic dysfunction that is not produced by coronary artery disease or conditions causing abnormal cardiac loading (valvular heart diseases, arterial hypertension, etc.). However, the existing definition includes a heterogenic spectrum of acquired and genetic disorders which generate both electrical and functional abnormalities. The DCM spectrum currently also includes the hypokinetic non-dilated phenotype, i.e., hypokinetic non-dilated cardiomyopathy (HNDC), to identify patients who are in an incipient phase of disease [1]. Early diagnosis is of uttermost importance especially in genetic disorders which have variable cardiac expression and account for more than one-third of DCM causes. This case report aims to highlight the role of multimodal imaging in DCM phenotyping.

## 2. Case Presentation

**Figure 1:**
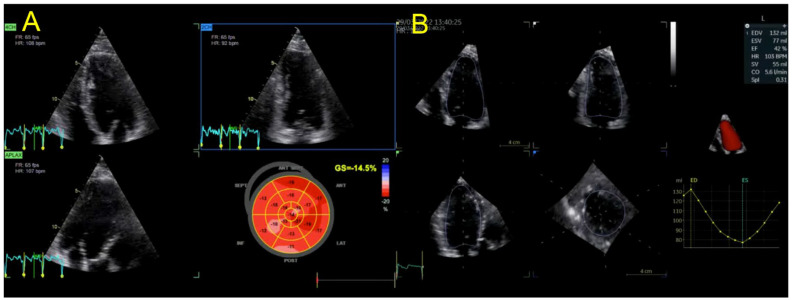
(**A**) 2DSTE LVGLS calculation and (**B**) 3DE LVEF and LV volumes in index patient. Abbreviations: 2DSTE—two-dimensional speckle-tracking echocardiography, LVGLS—left ventricular global longitudinal strain, 3DE—three-dimensional echocardiography, LV—left ventricular, LVEF—left ventricular ejection fraction.

**Figure 2:**
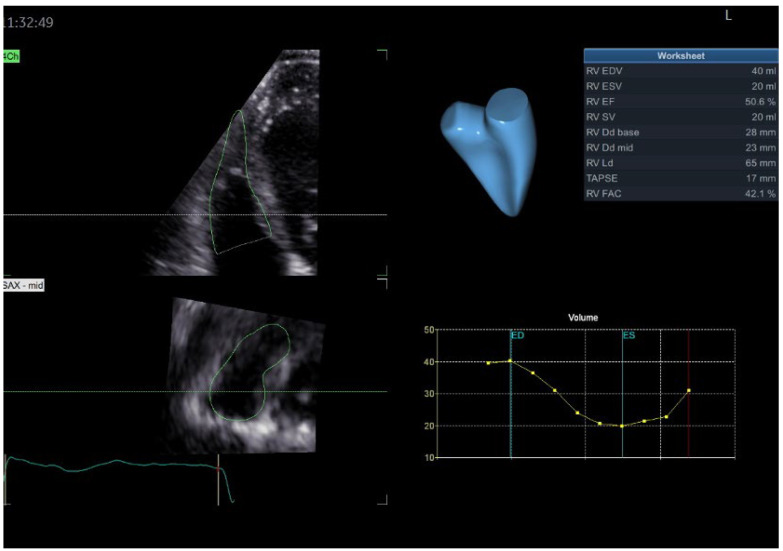
3DE RVEF and RV volumes in index patient. Abbreviations: 3DE—as in Figure 1, RV—right ventricular, RVEF—right ventricular ejection fraction.

We present the case of a 50-year-old male, with a personal history of grade 2 arterial hypertension and dyslipidemia who was sent to our clinical department for a routine check-up. A thorough familial history was obtained, revealing a deceased father at the age of 41 (due to presumed sudden cardiac death, SCD), a deceased brother at the age of 47 years old (SCD), and a 54-year-old brother diagnosed with heart failure of unknown etiology more than 10 years ago. Initially, we performed transthoracic echocardiography using both traditional and advanced techniques such as two-dimensional speckle tracking echocardiography (2DSTE) and three-dimensional echocardiography (3DE) (Figure 1), which exposed normal 3DE LV volumes, diffuse mildLV hypokinesia and systolic dysfunction, with a 3DE LV ejection fraction (LVEF) of 42%. The LV global longitudinal strain (LVGLS) was also reduced, with a value of −14.5%. Right ventricular (RV) function was normal, as quantified by the 3DE RV ejection fraction (RVEF) (Figure 2). An interatrial septum aneurysm was noted.

**Figure 3:**
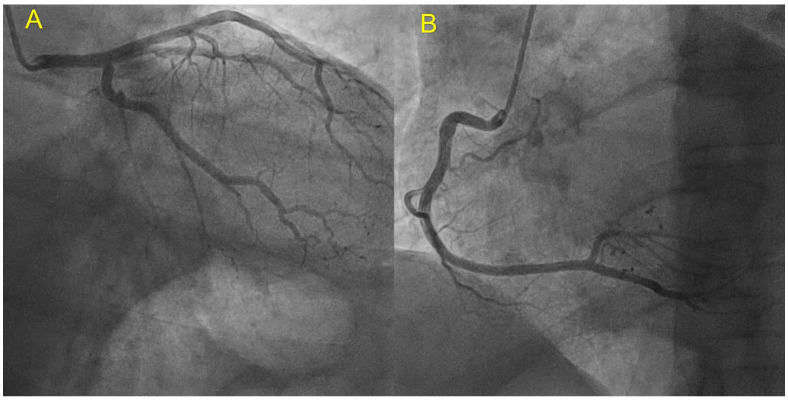
Coronary angiography of index patient showing normal coronary arteries. (**A**) Left coronary artery; (**B**) right coronary artery.

**Figure 4:**
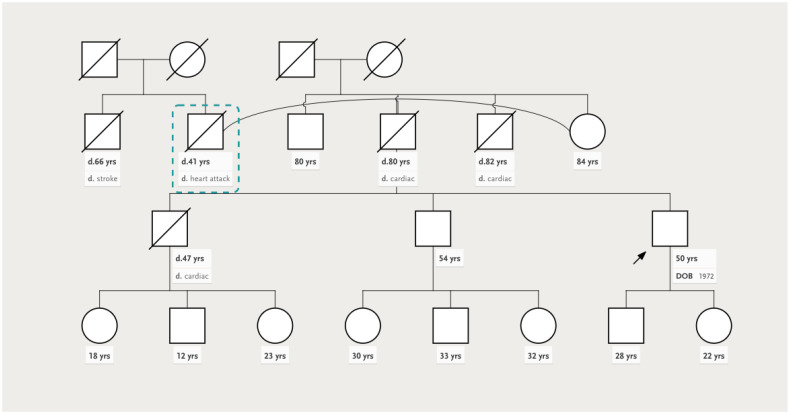
Familial pedigree. The arrow indicates the index patient. Abbreviations: d—death, yrs—years.

Subsequent evaluation consisted of coronary angiography which showed normal coronary arteries (Figure 3), ECG Holter monitoring (no arrhythmic events or conduction disturbances), and a complete familial pedigree (Figure 4).

Taking into consideration the familial history and echocardiographic aspect, we also decided on genetic testing. A heterozygous titin (TTN) variant was discovered, with a deletion in exon 326 in the A band of TTN. 

**Figure 5:**
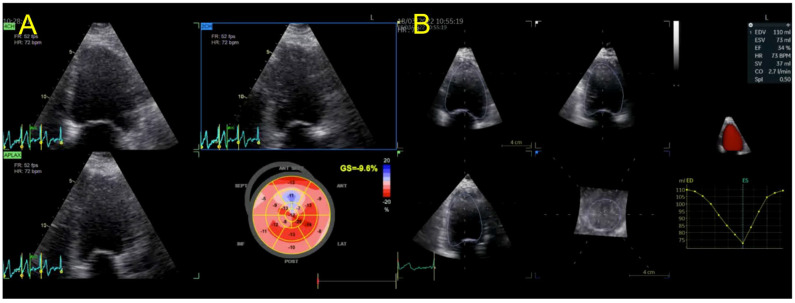
(**A**) 2DSTE LVGLS calculation and (**B**) 3DE LVEF and LV volumes in proband’s brother. Abbreviations: same as in Figure 1.

**Figure 6:**
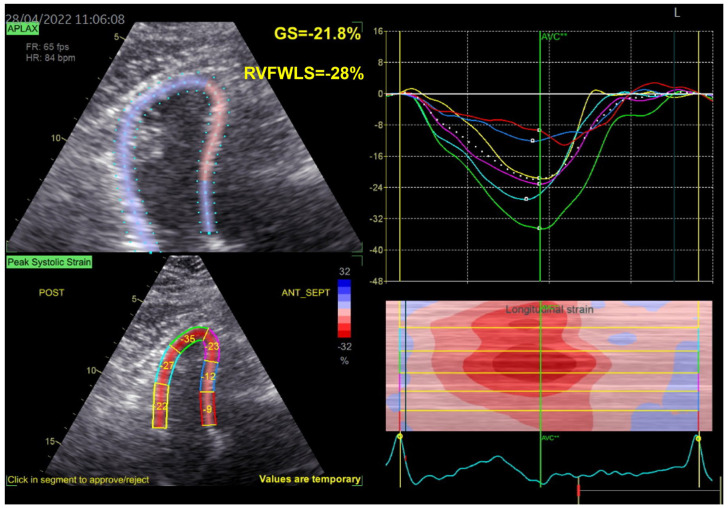
RVFWLS calculation in proband’s brother. We excluded the IVS for the calculation of RV strain and only used the free RV wall which was divided into three segments (basal, mid, apical) Abbreviations: RVFWLS—right ventricular free wall longitudinal strain, IVS—interventricular septum, RV—as in Figure 2.

We performed the same tests in the proband’s brother (who was 54 years old) and nephew (who was 33 years old). Similar echocardiographic findings were discovered in the proband’s brother, albeit with more advanced LV systolic dysfunction as observed both by 3DE LVEF and 2DSTE LVGLS (34% and −9.6%, respectively), and an important left atrial (LA) dilation (3DE LA maximum volume of 113 mL, i.e., 52 mL/m^2^) (Figure 5). RV function, determined only by right ventricular free wall longitudinal strain (RVFWLS), due to an inadequate echocardiographic window, was normal (Figure 6). Remarkably, an interatrial septum aneurysm was also present. 

**Figure 7:**
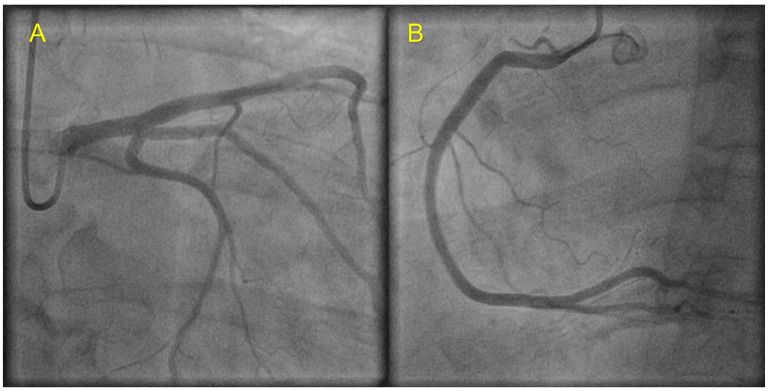
Coronary angiography of the index patient’s brother showing normal coronary arteries. (**A**) Left coronary artery; (**B**) right coronary artery.

The same TTN anomaly was present in the proband’s brother. Coronary angiography showed normal epicardial arteries (Figure 7).

**Figure 8:**
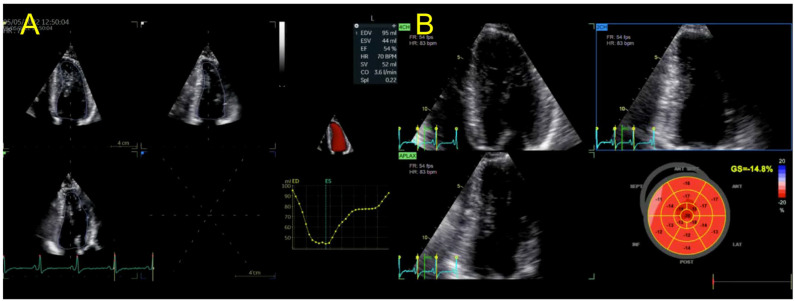
(**A**) 3DE LVEF and LV volumes and (**B**) 2DSTE LVGLS calculation in proband’s nephew. Abbreviations: same as in Figure 1.

The echocardiographic aspect of the third patient (the proband’s nephew, with hypertension, known for anabolic steroid use and resistance training) was that of a non-dilated LV with concentric hypertrophy, normal 3DE LVEF and mildly reduced LVGLS (−14.8%). RV function and volumes were in the normal range. No TTN variant was detected in this case. LV hypertrophy and LV sub-clinical systolic dysfunction (Figure 8) were interpreted in the context of arterial hypertension, resistance training and anabolic steroid use.

**Figure 9:**
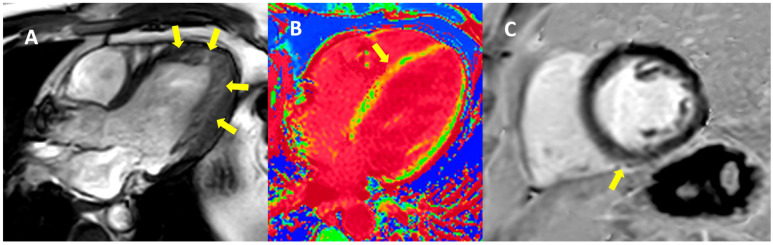
Cardiac magnetic resonance imaging. (**A**) SSFP cine image in 3-chamber view showing clear non-compaction of the LV in proband (arrow). (**B**) T1 colored mapping in 4-chamber view showing increased septal T1 values of ~1420 ms (yellow arrow) and (**C**) PSIR image in basal short-axis view showing possible small fibrosis in the inferior septum (arrow) in proband’s brother.

We continued by adjusting the medical treatment which in the case of the proband was necessary for better control of arterial hypertension, and in the case of the proband’s brother was modified as indicated by current guidelines for heart failure with a reduced ejection fraction (HFrEF). Both patients were scheduled for cardiac magnetic resonance (CMR) imaging three months later, which was performed on a 3.0 Tesla machine. In the proband’s case, long-axis Cine SSFP imaging showed a non-dilated LV while also demonstrating apical LV hypertrabeculation, meeting the criteria for LV non-compaction (LVNC; non-compacted/compacted myocardium = 2.7) (Video S1). Using short-axis Cine SSFP imaging, LV volumes and function were computed, showing normal LV volumes (LV end-diastolic volume—209 mL, i.e., 104 mL/m^2^; LV end-systolic volume—104 mL, i.e., 52 mL/m^2^), and mild LV systolic dysfunction (LVEF of 50%) due to discrete generalized hypokinesia. RV dimensions and function were normal. T2-weighthed imaging showed no signs of myocardial edema and T1 mapping sequences were normal. Late gadolinium enhancement (LGE) images were unequivocal. The CMR evaluation of the proband’s brother also showed a non-dilated LV with moderate LV systolic dysfunction due to diffuse hypokinesia (LV end-diastolic volume—193 mL, i.e., 89 mL/m^2^; LV end-systolic volume—113 mL, i.e, 52 mL/m^2^, and LVEF—41%) (Video S2). RV volumes were normal; however, a mild RV systolic dysfunction was detected (RV end-diastolic volume—162 mL, 75 mL/m^2^; RV end-systolic volume—87 mL, i.e., 40 mL/m^2^, and RVEF—46%). T2 mapping was normal, while T1 mapping sequences revealed high diffuse T1, especially at the level of the interventricular septum (IVS) (T1 = 1420 ms). LGE images showed sub-epicardial fibrosis at the junction between the inferior IVS and the RV, which was extended intramurally to the basal inferior wall (Figure 9).

Thus, we concluded that the aspect is that of HNDC, superimposed with LVNC in the probands’ case, and associated with biventricular involvement and myocardial fibrosis in the proband’s brother’s case (Table 1). In the future, we plan on evaluating the rest of the first-degree relatives of the proband and his brothers.

## 3. Discussion

This paper has two main highlights: (i) we identified a new titin variant mutation possibly involved in cardiomyopathies, and (ii) the association of hypokinetic non-dilated cardiomyopathy with left ventricular non-compaction. It also underlines the importance of a multimodal imaging approach in selected patients with different forms of cardiomyopathy in addition to a thorough check of personal and familial history and appropriate genetic testing [2]. 

Initial echocardiographic evaluation in the proband raised the suspicion of hypokinetic non-dilated cardiomyopathy. After excluding coronary artery disease as a potential causal factor, genetic testing using a 168-gene panel was performed, revealing a heterozygous TTN variant—deletion in exon 3 (exon 326, c.70482_70483del p.Tyr23494*)—which is expected to create a truncated TTN protein and that has not been previously reported in the literature in individuals affected by TTN-related conditions. CMR evaluation did not demonstrate myocardial edema, fatty infiltration, or fibrosis, but met the criteria for LVNC. The same TTN mutation was detected in the proband’s brother. The phenotype of the proband’s brother was also of hypokinetic non-dilated cardiomyopathy, exhibiting myocardial fibrosis upon LGE and biventricular involvement but without showing the criteria for LVNC. Finally, the proband’s nephew exhibited concentric left ventricular hypertrophy with normal LV function, considered secondary to exercise training and anabolic steroid use, but raising significant differential diagnostic difficulties given the familial aggregation of sudden cardiac death and cardiomyopathy with an identified mutation of titin.

According to a position statement of the ESC working group on myocardial and pericardial diseases, HNDC has emerged as a sub-type of DCM and is defined by LV or biventricular global systolic dysfunction without dilation (LVEF < 45%), while it is not explained by abnormal loading conditions or coronary artery disease [1]. The introduction of this new category is justified by the fact that in some cases, although patients exhibit LV systolic dysfunction with or without a known genetic cause, LV dilation is absent, which can lead to late or wrong diagnosis and treatment. According to a study by Guo et al., the prevalence of HNDC was estimated to be from 0.9% to 1.9%, slightly higher in women, and lower with increasing age [3]. It is considerably rarer than DCM and probably still underdiagnosed. Although compared to the dilated phenotype HNDC is associated with less advanced disease, higher LVEF and overall good clinical outcomes [4], it can evolve into DCM which worsens long-term prognosis (in up to 24% of cases, as found by Gigli and colleagues) [5]. Moreover, arrhythmogenic cardiomyopathy can have a non-dilated hypokinetic phenotype, which raises awareness once more regarding early disease recognition using multimodal imaging techniques [6]. In our clinical scenario, we cannot predict whether or not the overt phase of systolic disfunction will be associated with LV dilatation in the future (in which case the present phenotype would be just an intermediate stage of disease). On one hand, extrinsic unknown factors might promote dilation. On the other hand, guideline-adjusted treatment might slow or even prevent ventricular remodeling.

An important feature of our report is that the index case also showed positive criteria for LVNC upon cardiac magnetic resonance imaging. Left ventricular non-compaction is a relatively rare form of cardiomyopathy with variable classification. The American Heart Association considers it to be a genetically determined primary cardiomyopathy; however, in the classification of the European Society of Cardiology it is included in the category of unclassified cardiomyopathy. It is characterized by the presence of a myocardium with two distinct layers: a compact sub-epicardial layer and a non-compacted layer in the sub-endocardial area. This pathological layer is characterized by the presence of trabeculae with the delimitation of intertrabecular spaces (recesses) that communicate with the ventricular cavity. Non-compacted regions are preferentially located at the level of the lower and lateral walls of the left ventricle in the apical and middle segments, as well as at the apex. The clinical picture is variable—from asymptomatic patients to heart failure and sudden death. Major adverse cardiac events, mainly heart failure, ventricular arrhythmias, and sudden death in this setting, as in other forms of cardiomyopathy, including DCM, are strongly (inversely proportionally) correlated with LVEF and myocardial fibrosis upon LGE [7]. The diagnosis of cardiomyopathy by non-compaction is preferably echocardiographic, but there are uncertain situations in which a multimodal imaging approach is necessary, incorporating echocardiography, nuclear magnetic resonance, computed tomography, or ventriculography [8]. In these situations, CMR is the preferred technique. In addition to diagnostic purposes, its ability to define myocardial tissue characteristics makes it an optimal tool for risk stratification and prognosis.

From a genotypic viewpoint of the DCM syndrome, TTN mutations are the most frequent cause of genetic forms (20–25% of familial DCMs) [1] and are usually located at the level of the A band, with high penetrance after 40 years of age [9]. TTN is the largest protein in the human body and an essential component of the sarcomere. Its function is related but not limited to sarcomere integrity [10]. Studies show that TTN-truncating variants (TTNtv) promote peripartum cardiomyopathy and other forms of acquired cardiomyopathies, such as chemotherapy-induced ones [11]. The impact of TTN mutations in the development of cardiac disease is only partially understood to this day. Research is consistently being conducted to unravel the other ways in which TTN anomalies impact cardiac phenotypic disease expression [12]. In our case, a TTN mutation was detected, with incomplete penetrance, and was associated with a non-dilated phenotype of cardiomyopathy with certain particularities, which are described above. Non-compaction of the left ventricle also has genetic determinism, with sporadic and familial forms, related to the presence of mutations of sarcomeric (titin included), mitochondrial, Z-line or cytoskeletal proteins [13]. Phenotypic variability for the same mutation has been observed, suggesting the interplay of other factors (either genetic or environmental) that determine the clinical and imaging presentation of non-compaction. Patients with non-compaction may have left ventricular dilation, and their relatives may have dilated cardiomyopathy without meeting the criteria for non-compaction [14].

Specific fibrosis patterns have been discovered in different gene mutations responsible for DCM, and were also observed in our case, with basal inferior IVS fibrosis which was demonstrated in the proband’s brother and has been reported in TTN mutations. A presence of septal LGE has been associated with an increase in risk of death and SCD [15]. 

Regarding the arrhythmic and SCD risk related to TTN mutations, compared to mutations of genes encoding for proteins such as lamin A/C (LMNA, nuclear envelope protein), filamin C (FLNC, cytoskeletal protein), or phospholamban (PLN, calcium regulating protein) which have been associated with a malignant arrhythmic outcome, TTN mutations are presumably more benign. However, some studies point out that TTNtv do possess an arrhythmic burden. In a prospective study conducted on 572 DCM subjects, TTNtv remained predictive of early arrhythmias in a multivariate regression model, after excluding protein-altering LMNA variants [16]. A retrospective analysis of 148 DCM patients with an ICD/CRT-D device (in whom mutations of lamin A/C were an exclusion criteria) showed that a positive TTNtv status was linked to a higher risk of receiving appropriate ICD therapy for ventricular tachycardia (VT) or fibrillation (VF) especially when found to be combined with midwall fibrosis upon CMR [17]. However, although the family here reported has a history of sudden presumed cardiac death at a young age, given the lack of information, we cannot speculate the actual causes or the possible involvement of the TTN mutation.

## 4. Conclusions

This report highlights the importance of multimodal imaging in phenotyping different cardiomyopathies with or without familial genetic aggregation. The clinical spectrum of DCM includes a non-dilated phenotype which should not be ignored by clinicians as it can hide several pathogenic conditions. Although echocardiography remains the first-line imaging modality, CMR is superior especially in terms of tissue characterization, being able to detect myocardial fibrosis and certain morphological aspects even in the absence of LV dilation. Finally, when suited, genetic testing should be performed in selected patients, and extended to patients’ relatives, taking into consideration the complex phenotype–genotype interplay responsible for the development of disease. 

## Figures and Tables

**Table 1:** Summary of the findings.

	Proband	Brother	Nephew
**Clinical**	Arterial hypertension Dyslipidemia	NYHA II HFrEF Persistent atrial fibrillation Arterial hypertension	Anabolic steroid use Resistance training Arterial hypertension
**Echocardiography**	Hypokinetic non-dilated cardiomyopathy LVEF = 42% Mild mitral regurgitation IAS aneurysm	Hypokinetic non-dilated cardiomyopathy LVEF = 34% Mild mitral regurgitation IAS aneurysm	Left ventricular concentric hypertrophy LVEF = 54%
**Cardiac magnetic resonance imaging**	Hypokinetic non-dilated cardiomyopathy Left ventricular non-compaction LVEF = 50%	Hypokinetic non-dilated cardiomyopathy Regional sub-epicardial fibrosis LVEF = 41%	Not carried out
**Coronary angiography**	Normal	Normal	Not carried out
**Genetic testing**	Heterozygous TTN variant—deletion in exon 3 (exon 326, c.70482_70483del p.Tyr23494*)	Heterozygous TTN variant—deletion in exon 3 (exon 326, c.70482_70483del p.Tyr23494*)	No pathogenic/likely pathogenic mutations identified
**Management**	CV risk factor control HTN treatment optimization Clinical and echo follow up	CV risk factor control HFrEF treatment optimization Clinical and echo follow up after 3 months of OMT	CV risk factor control Cessation of steroid use Clinical and echo follow up

CV—cardiovascular; HFmEF—heart failure with mildly reduced ejection fraction; HFrEF—heart failure with reduced ejection fraction; HTN—arterial hypertension; IAS—interatrial septum; LVEF—left ventricular ejection fraction; OMT—optimal medical treatment; TTN—titin gene.

## Data Availability

Data are available upon request with the approval of the University of Medicine and Pharmacy of Craiova.

## Supplementary Materials

The following supporting information can be downloaded at: https://www.mdpi.com/article/10.3390/jpm13050742/s1, Video S1: SSFP Cine imaging in 3-chamber view showing LV non-compaction in proband; Video S2: SSFP Cine imaging in 3-chamber view showing a non-dilated LV with diffuse hypokinesia in proband’s brother.
